# Research progress on the pattern recognition receptors involved in porcine reproductive and respiratory syndrome virus infection

**DOI:** 10.3389/fcimb.2024.1428447

**Published:** 2024-08-15

**Authors:** Yulin Xu, Luogang Ding, Yuyu Zhang, Sufang Ren, Jianda Li, Fei Liu, Wenbo Sun, Zhi Chen, Jiang Yu, Jiaqiang Wu

**Affiliations:** ^1^ Shandong Key Laboratory of Animal Disease Control and Breeding, Institute of Animal Science and Veterinary Medicine, Shandong Academy of Agricultural Sciences, Jinan, China; ^2^ Key Laboratory of Livestock and Poultry Multi-omics of Ministry of Agriculture and Rural Affairs (MARA), Jinan, China

**Keywords:** PRRSV, PRRs, innate immune, PAMPS, signaling pathways

## Abstract

Porcine reproductive and respiratory syndrome (PRRS) is one of the most economically devastating infectious diseases of pigs globally. The pathogen, porcine reproductive and respiratory syndrome virus (PRRSV), is an enveloped positive-stranded RNA virus, which is considered to be the key triggers for the activation of effective innate immunity through pattern recognition receptor (PRR)-dependent signaling pathways. Toll-like receptors (TLRs), RIG-I-like receptors (RLRs), C-type lectin receptors (CLRs), NOD-like receptors (NLRs) and Cytoplasmic DNA receptors (CDRs) are used as PRRs to identify distinct but overlapping microbial components. The innate immune system has evolved to recognize RNA or DNA molecules from microbes through pattern recognition receptors (PRRs) and to induce defense response against infections, including the production of type I interferon (IFN-I) and inflammatory cytokines. However, PRRSV is capable of continuous evolution through gene mutation and recombination to evade host immune defenses and exploit host cell mechanisms to synthesize and transport its components, thereby facilitating successful infection and replication. This review presents the research progress made in recent years in the study of these PRRs and their associated adapters during PRRSV infection.

## Introduction

PRRS is a viral infectious disease that causes reproductive failure in pregnant sows and severe respiratory diseases in pigs of all ages. It is one of the most significant diseases affecting the global pig industry (Butler et al., 2014). The first documented cases of PRRS were reported in the Netherlands in 1991 and in the United States in 1992 (Wensvoort et al., 1991; Collins et al., 1992). PRRSV exhibits a number of unique characteristics, such as mutation and recombination, persistent infection, immunosuppression, and antibody-dependent enhancement of replication (Liu et al., 2017; Chen N. et al., 2018). The rapid evolution and mutation lead to the constant emergence of mutant strains and new strains (Guo et al., 2019). Furthermore, the humoral and cellular immune responses to PRRSV infection are delayed and low. Vaccination represents the primary strategy for the prevention and control of PRRS. However, the vaccines currently in use exhibit suboptimal immune effects and low immune protection rates, which preclude the achievement of complete control of the disease (Han et al., 2017). Therefore, the development of safe and effective vaccines has become a significant challenge in this field. In recent years, the search for host proteins that inhibit PRRSV replication has become the primary focus of anti-PRRSV research.

The innate immune system plays an important role in the process of host antiviral infection, acting as the host’s first line of defense against pathogen invasion. Pathogen associated molecular patterns (PAMPs) are recognized by host germline-encoded pattern recognition receptors, including Toll-like receptors (TLRs), RIG-I-like receptors (RLRs), NOD-like receptors (NLRs), C-type lectin receptors (CLRs), and cytosolic DNA receptors (CDRs) (Beutler, 2004; Mair et al., 2014; Chen et al., 2017). Upon activation, these receptors transmit to the signal adaptor protein, which then activates the expression of the transcription factors interferon regulatory factor 3/7 (IRF3/IRF7) and nuclear factor κB (NF-κB) through a series of signaling pathways. This induces the production of antiviral IFN-I and pro-inflammatory cytokines, collectively serving to resist infection by the pathogen (Liu et al., [[NoYear]]a; Liu et al., [[NoYear]]b).

## Toll-like receptors

TLRs are widely expressed in myeloid cells, including monocytes, macrophages, granulocytes, and dendritic cells (DCs). As host PRRs, they play an important role in the recognition of microbial PAMPs and the subsequent activation of specific signaling pathways that induce the transcription of inflammatory and/or anti-inflammatory cytokines (Zhou and Zhang, 2012; Schlaepfer et al., 2014). TLRs bind as homo- or heterodimers to the corresponding ligands on the plasma or organelle membrane and are mainly involved in downstream signaling via two adaptor proteins, Myeloid differentiation primary response protein 88 (MyD88) and Toll-interleukin-1 receptor (TIR)-domain-containing adaptor-inducing IFN-β(TRIF) (Gebremeskel et al., 2021; Liu et al., 2024). The signaling pathway of TLRs can be divided into two categories: the MyD88-dependent signaling pathway and the MyD88 non-dependent signaling pathway, which is based on the identified adaptor proteins. Upon stimulation by an external signal, the TIR domain at the C-terminal of the adaptor protein binds to the TIR domain of TLRs to form a complex. This complex is then recruited by the death domain (DD) at the N-terminal of MyD88, which binds to the signaling protein of the IL-1R-related kinase (IRAKs) family to activate NF-κB transported into the nucleus and induce the expression of pro-inflammatory cytokines (Tapping, [[NoYear]]; Yang and Seki, [[NoYear]]).

There have been several reports on the correlation between TLR and PRRSV infection. Infection with PRRSV *in vivo* tended to up-regulate the mRNA expression of TLR2, 3, 4, 7, and 8 in at least one of the lymphoid tissues, suggesting that TLR-mediated innate immunity likely plays a critical role in the pathogenesis of PRRSV infection in pigs (Liu et al., 2009). In contrast, the downregulation of TLR7 and 8 by poly(I:C) stimulation and PRRSV infection was observed in both PAMs and immature DCs (Chaung et al., 2010). However, when the TLR3 signaling pathway was activated by poly (I:C), the viral load of PRRSV was significantly reduced, while the expression of TLR3 was inhibited, resulting in enhenced infectivity of PRRSV (Sang et al., 2008; Kuzemtseva et al., 2014). The TLR7 ligand (SZU101) can prevent the replication of PRRSV (Huang et al., 2022). Therefore, TLR3 and TLR7/8 ligands are promising adjuvant candidates for the development of novel vaccines against PRRSV (Zhang et al., 2013a). In a separate study, it was observed that highly pathogenic PRRSV infection induced higher expression levels of TLR3, 7, and 8 mRNA in PAMs and cerebral medullar tissues than low pathogenic PRRSV, indicating that the TLR expression levels were correlated with PRRSV virulence (Zhang et al., 2013b). Another report indicated that the PRRSV 3’ UTR pseudoknot region could act as PAMPs recognized by RIG-I and TLR3 to induce IFN-I production to suppress PRRSV infection (Xie et al., 2018). TLR4 has long been thought to be involved in innate immunity, mediating inflammatory responses by recognizing lipopolysaccharide (LPS) or bacterial endotoxins (Zhang et al., 2022). However, the study found that LPS inhibits PRRSV infection by down-regulating the expression of CD163 through the TLR4-NF-κB pathway (Zhu et al., 2020). PRRSV infection can also induce IL-1β maturation by activating the Nucleotide-binding oligomerization domain (NOD)- leucine-rich repeat (LRR)-and pyrin domain-containing protein 3 (NLRP3) inflammasome through the TLR4-MyD88-NF-κB pathways and then inhibit the proliferation of classical swine fever virus (Chen et al., 2023). In addition, the adaptor protein MyD88 was found to be involved in PRRSV-induced IL-1β production in microglia (Chen et al., 2018) and to play an important role in IL-10 induction during PRRSV infection (Song et al., 2013).

## RIG-like receptors

RLRs including retinoic acid-induced gene I (RIG-I), melanoma differentiation associated gene 5 (MDA5) and laboratory of genetics and physiology 2 (LGP2), recognize viral or other xenogeneic nucleic acid in the cytoplasm (Loo and Gale, 2011). RIG-I and MDA5 consist of caspase activation and recruitment domains (CARD), the DExD/H-box helicase domain and a C-terminal domain (CTD) (Yoneyama et al., 2004; Yoneyama et al., 2005). Activation of RIG-I or MDA5 interacts via CARD-CARD interactions with mitochondrial antiviral signaling (MAVS) protein (Seth et al., 2005). These interactions facilitate the relocation of RLRs to mitochondria membranes and the formation of MAVS signalosome with the downstream signaling molecules TANK-binding kinase 1 (TBK1) and IκB kinase-ϵ (IKK-ϵ). This complex phosphorylates and activates IRF3 and IRF7 (Moore and Ting, 2008), which in turn initiate the transcription of a variety of cytokines to resist virus infection. MAVS also recruits the Fas-associated death domain protein (FADD), which activates caspase-10 and caspase-8, driving NF-κB activation (Kawai and Akira, 2007). Upon activation, IRF3/7 and NF-κB translocate to the nucleus to trigger the transcription of a variety of cytokines, which are responsible for resisting virus infection. In comparison to RIG-I and MDA5, LGP2 has helicase and regulatory domains, but lacks the N-terminal CARDs, which are essential for antiviral signal transduction (Zhu et al., 2014). Consequently, LGP2 is unable to initiate the downstream signaling pathway independently and requires binding to RIG-I or MDA5 for signal transduction.

Furthermore, there are associations between PRRSV and members of RLR family. It has been reported that the overexpression of LGP2 can inhibit PRRSV replication. PRRSV Nsp1 and Nsp2 interacted with LGP2 and promoted K63-linked ubiquitination of LGP2, which ultimately led to the degradation of LGP2 (Zhu et al., 2023). The endoribonuclease activity of PRRSV Nsp11 is critical for both viral replication and the inhibition of IFN-I production. Of note, Nsp11 suppresses both MAVS and RIG-I expression to antagonize IFN-I production (Sun et al., 2016). In addition, studies have shown that endogenous expression of the porcine 2’, 5’-oligoadenylate synthetase (OAS2) gene can be enhanced by interferon IFN-β or PRRSV infection in porcine alveolar macrophages (PAMs). Additionally, the porcine OAS2 has been shown to suppress the replication of PRRSV in a RIG-I-dependent manner (Zhao et al., 2017). And the N protein of PRRSV interferes with TRIM25-mediated RIG-I ubiquitination to suppress the host innate immune response (Zhao et al., 2019).The study indicated that MAVS is involved in the interaction between PRRSV and the host immune response and that MAVS mediates a powerful anti-PRRSV process (Zhao et al., 2019). However, Nsp4 of HP-PRRSV has been demonstrated to cleave MAVS to impair antiviral responses mediated by RIG-I-like receptors (Huang et al., 2016). And PRRSV infection promotes glycolysis to produce lactate, which targets MAVS to inhibit RLR signaling and thus promote viral replication (Zhang et al., 2023).

## NOD-like receptors

The NLR pathway is also instrumental in the detection of cytosolic DNA and the initiation of inflammation-dependent innate immune signals (Petrilli et al., 2007). Some NLRs are sensitive to many PAMPs and release the inflammatory cytokines of the IL-1 family via caspase-1, including IL-1β, IL-18 and IL-33 (Meylan et al., 2006; Yu and Finlay, 2008). The inflammatory bodies of NLR protein can be divided into three main types: the NALP3 inflammasome (also known as the NLRP3 inflammasome), the NALP1 (NLRP1) inflammasome and the IPAF (NLRC4) inflammasome (Kawai and Akira, 2009). These inflammasomes involve an adaptor—apoptosis-associated speck-like protein containing a CARD (ASC), which links these NLRs to caspase-1 (Kawai and Akira, 2009). Furthermore, all NLRs except NOD1 and NOD2 activate inflammasome formation independently of gene transcription through oligomerization of the adaptor ASC, which induces maturation of IL-1β and IL-18 (Hoss et al., 2017). In contrast to other NLRs, the activation of NOD1 and NOD2 recruits a common downstream signaling adapter, RIPK2, which in turn activates downstream NF-κB and the production of pro-inflammatory cytokines (He and Wang, 2018). Studies have shown that RIPK2 is involved in the activation of NF-κB and MAPK (McCarthy et al., 1998), while porcine RIPK2 does not activate IRF3 (ISRE) activity (Ao et al., 2020). It has been demonstrated that ASC functions as a dual regulator of NF-κB, whereas ectopic ASC is only a weak activator of NF-κB (Stehlik et al., 2002).

DDX19A, a member of the DEAD/H-box protein family, was identified as a novel component of NLRP3 inflammasome. The research found that DDX19A interacted with PRRSV RNA and NLRP3. Knockdown of DDX19A expression efficiently inhibited procaspase-1 cleavage and IL-1β secretion in PRRSV-infected or PRRSV RNA-stimulated PAMs (Li et al., 2015). NLRP3 inflammasome is activated by PRRSV in microglia, which is required for IL-1β secretion (Chen et al., 2018), but the virus protein Nsp11 can inhibit this effect (Wang et al., 2015). PRRSV infection increased the expression of NOD2, NLRP3 and RIP2 and enhances phosphorylation of RIP2 (Jing et al., 2014). NLRX1, a member of the NLR family proteins, is initially identified as key mediators of immune defense and inflammation (Tattoli et al., 2016).The LRR domain of NLRX1 could interacted with the RNA-dependent RNA polymerase (RdRp) domain of Nsp9 to inhibit PRRSV replication (Jing et al., 2019).

## CLR like receptor

CLR is comprised of over 1,000 receptors, including soluble or membrane-bound receptors, which are distinguished by the presence of at least one carbohydrate recognition domain (CRD) or C-type lectin-like domain (CTLD) (Zelensky and Gready, 2005). It is well established that CLRs, including Dectin-1, Dectin-2, Dectin-3 and Mincle, play an important role in the host defense against fungal infections by using their CRD to recognize the cell wall component from the infected microorganisms (Shiokawa et al., 2017; Tang et al., 2018). Upon recognition of their carbohydrate agonists, they induce activation of the immunoreceptor tyrosine activating motif (ITAM)-like motif (Hem ITAM) in Dectin-1 or ITAM in the interaction adapter FCRγ of Dectin-2, Dectin-3 and Mincle via Src family kinases. The splenic tyrosine kinase (Syk) was recruited and phosphorylated by activated Hem-TAM/ITAM, and the phosphorylated Syk then triggered the formation of the caspase recruitment domain complex, which included caspase recruitment domain 9 (CARD9), B-cell lymphoma/leukemia 10 (BCL10) and mucosa-associated lymphoid tissue 1 (MALT1). The CARD9-BCL10-MALT1 (CBM) adaptor complex or signalosome subsequently activates the NF-κB pathway through various mechanisms (Ruland and Hartjes, 2019), and produces the cytokines IL-1β, IL-6, and IL-23 (Tang et al., 2018).

The CBM signaling components are highly conserved in evolution and have been extensively studied in humans and mice, and identified in ray-finned fishes (Staal et al., 2018; Ruland and Hartjes, 2019). CARD9, one of the receptors of CLRs, is expressed exclusively in myeloid cells (Gross et al., 2006; Hara et al., 2007). BCL10 is the central adaptor protein whose amino-terminal CARD mediates homologous interaction with CARD9, CARD10, CARD11 or CARD14 (Bertin et al., 2001; Wang et al., 2001; Ruland and Hartjes, 2019). In our previous study, CARD9 was found to be essential for CBM maximal signaling, although it is not constitutively active. Furthermore, BCL10 and MALT1 exhibited optimal synergism when present in moderate amounts of BCL10 and low amounts of MALT1 (Jiang et al., 2020). Among these, MALT1 is rapidly induced upon PRRSV infection and mediates the degradation of two anti-PRRSV RNases, MCPIP1 and N4BP1, relying on its proteolytic activity to facilitate PRRSV replication. Several PRRSV Nsps, including Nsp11, Nsp7β, and Nsp4, has been shown to contribute to MALT1 induction. Finally, PRRSV Nsp6 was found to mediate significant MALT1 degradation via the ubiquitination-proteasome pathway (Gu et al., 2022).

## CDR like receptors

CDRs, such as cGAS and IFI16, utilize the adaptor protein STING to activate the expression of transcription factors IRF3/IRF7 and NF-κB, subsequently inducing the downstream production of antiviral IFN-I and proinflammatory cytokines. The innate DNA receptor cGAS is capable of recognizing both self and non-self double-stranded DNA. It is a cyclic GMP-AMP(cGAMP) synthase, and catalyzes the synthesis of 2’3’-cGAMP from ATP and GTP. Subsequently, 2’3’-cGAMP, as a second message, binds STING on endoplasmic reticulum (ER) and triggers the STING translocation from ER. Next, the STING recruits TBK1 which activates IRF3 and NF-κB transcriptions (Sun et al., 2013; Hopfner and Hornung, 2020). The gene transcriptions result in the generation of downstream IFNs, IFN-stimulated genes (ISGs), and proinflammatory cytokines, which play an important antiviral role in virus infections (Sun et al., 2013; Hopfner and Hornung, 2020). STING was initially identified as an IFN-stimulating factor and is widely expressed in a variety of tissues and cells, suggesting that it may have a function in immune regulation (Bo et al., [[NoYear]]; Ishikawa and Barber, [[NoYear]]). Furthermore, STING plays a crucial role in mediating the innate immune response to viruses, intracellular bacteria and even intracellular parasites. Additionally, it is involved in the IFN-I signal pathway which is initiated by pathogenic RNA viruses, through its interaction with the adaptor protein MAVS. It has been demonstrated that the host cGAS-STING signaling pathway plays an important antiviral role in PRRSV infection (Xu et al., 2021). cGAS can restrict PRRSV replication by sensing the mtDNA in the cytoplasm by increasing cGAMP activity (Liu et al., 2022). Recent research has shown that astragaloside IV has the capacity to mitigate the adverse effects of PRRSV infection on innate immune function, reactive the inhibited cGAS-STING signaling pathway, and enhance the expression of IFN-I, ultimately exerting antiviral effects (Song et al., 2023). In addition, PRRSV Nsp2 retains STING in the ER by increasing Ca2^+^ sensor stromal interaction molecule 1 (STIM1) protein level and deubiquitinates STIM1 through its papain-like protease 2 (PLP2) deubiquitination activity to limit PRRSV replication (Diao et al., 2023).

IFI16, a member of the PYHIN protein family containing a pyrin domain and two DNA-binding HIN domains, displays diverse activity due to its ability to bind to various target proteins and modulate various functions including direct actions in regulation of transcription, proliferation, differentiation, apoptosis, antiviral restriction, and inflammation (Gariano et al., 2012; Orzalli et al., 2012; Jakobsen and Paludan, 2014). IFI16 has been reported to play a broader role in the regulation of interferon-stimulated gene expression, leading to responses not only to DNA viruses, but also to RNA viruses such as Sendai virus (Unterholzner et al., 2010; Thompson et al., 2014). Overexpression of IFI16 could significantly suppress PRRSV-2 replication, and silencing of endogenous IFI16 expression by small interfering RNAs resulted in the promotion of PRRSV-2 replication in MARC-145 cells. Toosendanin inhibits PRRSV-2 via an IFI16-dependent pathway (Zhang et al., 2022). In addition, IFI16 could promote MAVS-mediated IFN-I production and interact with MAVS. More importantly, IFI16 exerted anti-PRRSV effects in a MAVS-dependent manner (Chang et al., 2019).

## Perspectives and conclusion

**Figure 1:**
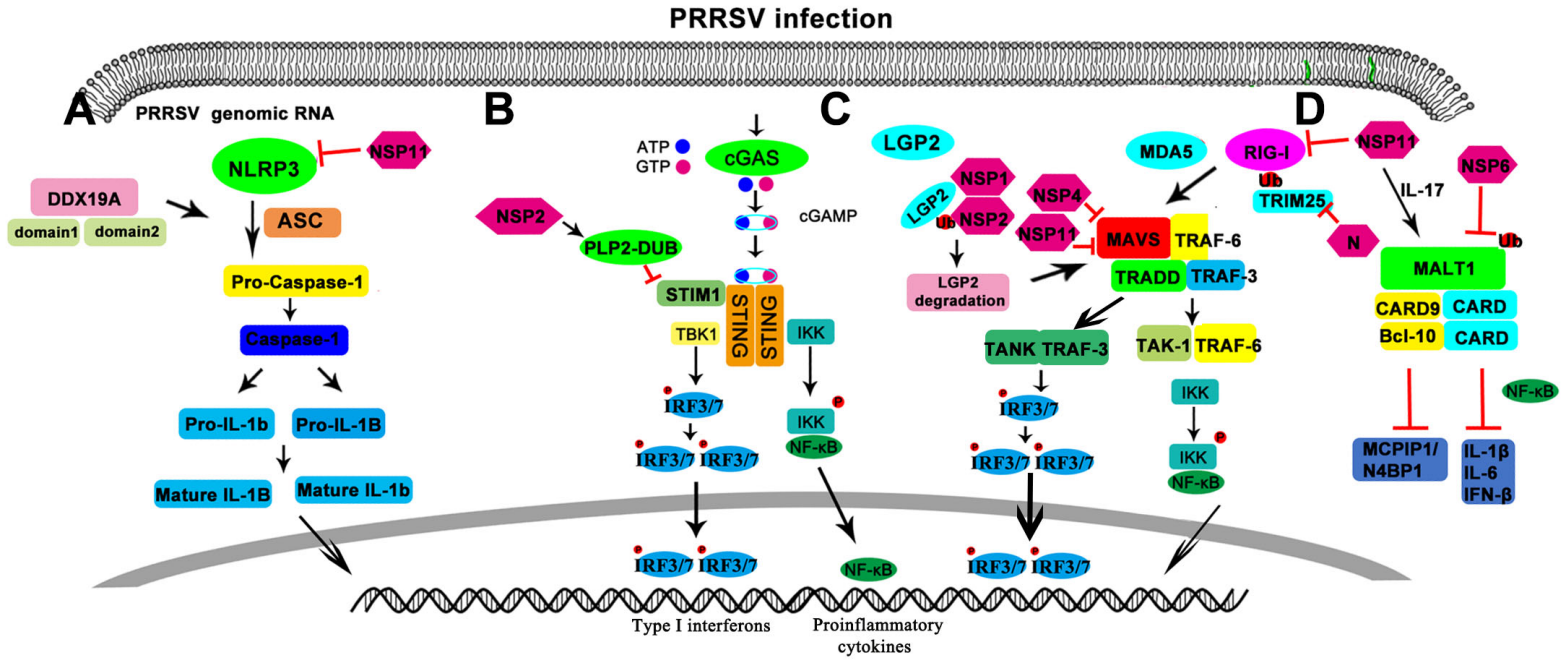
Schematic diagram of regulation of the pattern recognition receptors in PRRSV infection. **(A)** NOD-like receptors. **(B)** CDR like receptors. **(C)** RIG-like receptors. **(D)** CLR like receptor.

The discovery of transmembrane TLRs and cytoplasmic sensing systems (such as RLRs, NLRs and CDRs) shows that the innate immune system has many recognition mechanisms in different cellular compartments (such as plasma membrane, endosome, lysosome, cytoplasm) and different cell types (such as TLR7 and TLR9 in pDCs and RLR in cDCs) (Kawai and Akira, 2009). Indeed, the relationship between signal adaptors is complex. For example, STING enhanced the activity of MAVS and NF-κB, while MyD88 enhanced the activity of STING ISRE. TIRAP-MyD88 (TLR2 and TLR4 signaling) and TRAM-TRIF (TLR4 signaling) induce inflammatory responses by recruiting TRAF6 (a member of the TRAF protein family) (Kawai and Akira, 2009). MyD88 recruits TRAF3 through TLR7 or TLR9 signaling and TRIF3 recruits TRAF3 in TLR3 signaling, all of which induce IFN-I (Hacker et al., 2006; Oganesyan et al., 2006). TRAF3 is also involved in RLR-mediated IFN-I induction (Saha et al., 2006). In addition, the RLR RIG-I directly interacts and cross-interferes with NOD2 (Morosky et al., 2011). ASC has been shown to interact with MAVS and inhibit MAVS-induced IFN-β production (Han et al., 2018). NLRC3, a member of the NLR protein family, has been shown to be a negative regulator of innate immune signals induced by the DNA sensor STING (Zhang et al., 2014). CARD9 is also involved in TLR, NOD1 and NOD2 mediated MAPK activation (Kawai and Akira, 2009). It appears that each PRR signaling pathway plays an essential role in pathogen elimination or tolerance maintenance (**Figure 1**).

Over the past decade, although scientists from around the world have conducted more research on PRRS and its related signaling pathways, people’s understanding of the pig innate immune signaling pathway is not clear enough. In particular, there are many innate immune signal adaptors and their signaling pathways, making it difficult to have a comprehensive understanding. Therefore, we still need to better understand the dynamics and breadth of innate immunity in tissues-, species- and host-specific manners. Although more adaptor proteins have been found in PRRS viruses, their role in the pathogenesis of PRRS has not been clearly elucidated. Therefore, there is a lack of research and understanding of the effect of swine innate immunity on PRRSV replication and its mechanism. Filling these gaps will provide new ideas for the in-depth study of the molecular mechanisms by which the host recognizes and clears PRRSV infection and by which PRRSV evades the host immune system. In summary, we have reviewed and described the roles of PPRs in host defense against PRRSV infection and proposed areas of research that require further investigation. With the development and application of new immunological approaches available in swine, new insights into the PPRs against PRRSV after infection will be discovered. This will facilitate the development of vaccines against PRRSV and improve our understanding of antiviral immunity in swine.
